# Thrombotic thrombocytopenic purpura - analysis of clinical features, laboratory characteristics and therapeutic outcome of 24 patients treated at a Tertiary Care Center in Saudi Arabia

**DOI:** 10.12669/pjms.326.11274

**Published:** 2016

**Authors:** Shahid Iqbal, Syed Z. A. Zaidi, Ibraheem H Motabi, Nawal Faiez Alshehry, Mubarak S. AlGhamdi, Imran Khan Tailor

**Affiliations:** 1 Shahid Iqbal MBBS, FCPS, MRCP (UK), Department of Adult Hematology / BMT, CCC, King Fahad Medical City, Riyadh, Saudi Arabia; 2 Syed Z. A. Zaidi, MBBS, FRCP, FCPS, FRCPath, FACP, Department of Adult Hematology / BMT, CCC, King Fahad Medical City, Riyadh, Saudi Arabia, Faculty of Medicine, King Saud Bin AbdulAziz University of Health Sciences, Riyadh, Saudi Arabia; 3 Ibraheem H Motabi, MRCP, MD, Department of Adult Hematology / BMT, CCC, King Fahad Medical City, Riyadh, Saudi Arabia, Faculty of Medicine, King Saud Bin AbdulAziz University of Health Sciences, Riyadh, Saudi Arabia; 4 Nawal Faiez Alshehry, MD, SBIM, SBAH, Department of Adult Hematology / BMT, CCC, King Fahad Medical City, Riyadh, Saudi Arabia; 5 Mubarak S. AlGhamdi, MD, Department of Adult Hematology / BMT, CCC, King Fahad Medical City, Riyadh, Saudi Arabia; 6 Imran Khan Tailor, MBBS, MD, MRCP, FRCPath, Department of Adult Hematology / BMT, CCC, King Fahad Medical City, Riyadh, Saudi Arabia

**Keywords:** Thrombotic thrombocytopenic purpura (TTP), therapeutic plasma exchange (TPE), Rituximab

## Abstract

**Objective:**

Thrombotic thrombocytopenic purpura (TTP) is a life-threatening disease. The primary aim was overall response rate (ORR) assessment in the treated patients

**Methods:**

This retrospective study included 24 patients treated during 2006-2015. TTP patients with microangiopathic hemolysis (MAHA) and thrombocytopenia were included. We analyzed clinical features, laboratory characteristics and treatment outcomes of 24 TTP patients treated at our tertiary care center (KFMC).

**Results:**

Twenty-four TTP patients (18 females; 6 males) had a mean age of 33.5±13.9 years; 22(91%) had neurologic features, 7(29%) fever, 10(42%) renal impairment; 4(20.83%) cardiac manifestations; 22(91.7%) had triad with additional neurologic abnormalities; only 2(8.2%) had pentad of TTP. Majority (54.16%) had idiopathic TTP. All patients received therapeutic plasma exchange (TPE); 23(95.8%) received adjunctive corticosteroids and 13(54.2%) received rituximab either due to refractoriness to TPE on ~day7, or earlier. Twenty-one out of 24 (87.5%) achieved complete remission (CR) without any subsequent relapse. At 22 months (median, range 1-113), 20 patients (83.3%) are alive at the time of report. Three patients died during acute episode because of sever disease or delayed treatment and one died in CR.

**Conclusion:**

TPE, steroids and or rituximab was very effective in preventing high risk of mortality and achieving durable CR in 87.5% of patients. More awareness is needed for early diagnosis and early referral to centers with appropriate tertiary care facilities..

## INTRODUCTION

Thrombotic thrombocytopenic purpura (TTP) is a rare, life-threatening disease characterized by microvascular platelet deposition and thrombus formation with resulting microangiopathic hemolytic anemia (MAHA), thrombocytopenia is often accompanied by fever, renal failure, and neurological deficits. Deficiency of the von-Willebrand factor (vWF) cleavage metalloprotease, also known as ADAMTS13, has been implicated as an important etiological factor in TTP. Although congenital TTP cases exist, most sporadic cases of TTP appear to be associated with severe deficiency of ADAMTS13 activity due to autoantibodies against it.^1^ After endothelial damage, huge amounts of vWF stored as ultra-large multimers (ULMM) in Weibel Pallade bodies, is released in the circulation. Due to deficiency of ADAMTS-13, ULMM can’t be spliced to useful sized vWF-multimers. ULMM attract platelets in circulation to form platelet thrombi. Consumption of platelets in platelet-rich thrombi results in thrombocytopenia (Platelets usually 10-30x10^9/l) and these thrombi appear to be responsible for the renal and cerebral lesions, often damage other organ systems.^2^-^5^

Acute morbidities include stroke, transient ischemic attacks, myocardial infarction, arrhythmia, bleeding, and azotemia. TTP during pregnancy may precipitate fetal loss.^1^ Therapy should be initiated if the diagnosis of TTP is suspected on the basis of MAHA and thrombocytopenia (even in the absence of fever, CNS and Renal manifestations) within 4-8 hours.^6^ TTP was often fatal with a mortality rate exceeding 90% before total plasma exchange (TPE) was introduced with a response rate of ~80% and a survival rate >90%, indicating that early and rapid diagnosis can ameliorate the prognosis in TTP.^7^,^8^ TPE and immunosuppressive therapies remain the mainstay of treatment.

Few small reports have been published about Saudi patients with TTP until now.^9^-^11^ In the present study, we analyzed the clinical and laboratory characteristics, complications, therapy and outcome of 24 TTP patients treated at our large tertiary care center.

## METHODS

In this retrospective study, after approval of our Institutional review board (IRB), data of 24 patients of TTP (treated from October 2006 to April 2015) with features of MAHA and thrombocytopenia were identified by review of records of King Fahad Medical City (KFMC). The diagnostic criteria were based on: (1) thrombocytopenia (<100 ^109/L) without other identifiable causes; (2) MAHA with schistocytes on the peripheral blood smear; and (3) high LDH. Other thrombotic microangiopathies were excluded, including DIC, cancer and preeclampsia.

### Methods

Routine laboratory tests such as peripheral blood cell counts, reticulocyte count, coagulation profile, serum lactate dehydrogenase (LDH), bilirubin, serum creatinine, cardiac enzymes, and urinalysis, were performed. Peripheral blood smears were routinely prepared from all TTP patients and frequency of the schistocyte on the blood film was estimated as mild, moderate and severe at 1000X magnification. Wherever logistically possible ADAMTS13 levels and inhibitor titer were determined.

### Statistical Analysis

Baseline demographic characteristics were calculated in frequencies and percentages. All continuous variables were expressed as Mean±SD, or median and ranges. The primary aim was outcome assessment by ORR in the treated patients and remission status over follow-up period through Kaplan-Meier method. Paired sample t-test was applied to determine the mean significant difference among platelets (Plt), hemoglobin (Hgb) & LDH on Day 1 and Day 7 of treatment. P value < 0.05 was considered as statistically significant. All data were entered and analyzed through statistical package SPSS version 22.

## RESULTS

Twenty-four TTP patients included 18 females and 6 males. The mean age was 33.5±13.9 years. Twenty-two (91%) of the patients presented with neurologic features (Table-I). Twenty-two patients (91.6%) had the triad of hemolytic anemia, thrombocytopenia and neurologic abnormalities and only 2 (8.3%) had the classical pentad of TTP (Table-I). Among the plausible etiology, idiopathic (54.16%) was the most common followed by autoimmune abnormalities (29.2%had SLE. Moderate to severe schistocytosis was present in all cases and other laboratory features are shown in Table-I. Anti-ADAMTS13 IgG antibodies could be tested in 9 patients. Six of 9 patients (66.6%) were positive for anti-ADAMTS13 IgG antibodies (Range of Titer 0.4-25 BU).

**Table-I:** Characteristics of the 24 TTP patients.

Characteristics	
Age (Range), median	33.5 (17-63)
*Gender*	
Male	N=6 (25%)
Female	N=18 (7 5%)
Neurological manifestations	N=22 (91.7%)
• Headache	N=5 (20.8%)
• Confusion	N=6 (25.0%)
• Psychiatric symptoms	N=4 (16.7%)
• Dizziness	N=6 (25.0%)
• Seizures	N=5 (20.8%)
• TIA	N=3 (12.5%)
• Coma	N=1 (4.2%)
• Stroke	N=6 (25.0%)
Fever	N=7 (29.1%)
Renal manifestations	N=10 (41.7%)
Patients with triad (MAHA+Neurological+thrombocytopnia)	N=22 (91.6%)
Patients with TTP pentad	N=2 (8.3%)
Cardiac manifestations	N=4 (16.6%)
Symptomatic Thrombocytopenia	N=11 (45.8%)
Hemoglobin g/dl Median(Range)	8.25 (4.08-13.6)
Platelets x10^9/l Median(Range)	14 (5 - 57.9)
LDH IU/L Median(Range)	981 (186-4413)
Bilirubin Total umol/l Median(Range)	30.4 (3.6-127)
Bilirubin Indirect umol/l Median(Range)	24.3 (2.6-87)
Creatinine umol/l Median(Range)	84 (44-699)
*ADAMTS13 activity %*	
• <5% (very severe deficiency)	N=8 (42.1%)
• 5% - 10% (severe deficiency)	N=5 (26.3%)
• 10% - 50% (Low)	N=3 (15.8%)
• > 50% (Normal)	N=3 (15.8%)
*Plausible Etiological Factors:*	
• Idiopathic	N=13 (54.16%)
• SLE	N=6 (25%)
• Pregnancy + SLE	N=1 (4.16%)
• Pregnancy	N=2 (8.33%)
• Malignancy (multiple myeloma)	N=1 (4.16%)
• Congenital + Pregnancy	N=1 (4.16%)
Follow-up time months’ median(range)	22 (1-113)

On Day1 and Day7 of TPE, Median platelet count, hemoglobin and LDH had significant differences (P value< 0.001, < 0.007 and < 0.001 respectively) (Table-II).

**Table-II:** Paired comparison analysis of Platelet count, Hemoglobin and LDH on Day1 and Day7 of TPE.

Parameters	Minimum	Maximum	Mean±S.D	P - values
Plt count Day1 of TPE	5.00	57.90	18.77±14.13	*< 0.001
Plt count Day7 of TPE	22.00	419.00	155.92±112.8	
Hgb D1 of TPE	4.08	13.60	8.21±2.01	*0.007
Hgb D7 of TPE	7.26	12.90	9.52±1.38	
LDH D1 of TPE	186.00	4413.00	1211.17±971.41	*< 0.001
LDH D7 of TPE	131.00	463.00	278.92±87.93	

* means p value is significant.

On admission, 10(41.7%) patients required ICU support. All patients received TPE whereas 23(95.8%) patients received adjunctive corticosteroids. Five (20.8%) were early refractory to standard treatment with TPE. Thirteen (54.16%) patients received rituximab either due to refractoriness to TPE on ~Day5-7, or earlier due to cardiac or neurological manifestations at treating physician’s discretion. Average hospital stay was 27 days (range 1-131).

Median number of TPE sessions in relation to Rituximab usage is shown in Table-III. Four patients (16.7%) developed infection with different organisms and 3(12.5%) developed line related complications.

**Table-III:** Therapy details, complications and outcome of 24 patients with TTP.

Therapy details, complications and outcome	Median (ranges) Number (%)
Number of TPE	15.5 (1–52)
Inpatient days	27 (1–131)
Follow up time	22 (1–113)
Average plasma volumes	1.2 (1–1.5)
Early refractory	5 (20.8%)
Steroids Received	23 (95.8%)
Rituximab Received	13 (54.2%)
• Rituximab 1000 mg (in SLE patient)	1 (4.2%)
• Rituximab 375 mg/m^2	12 (50.0%)
ICU needed	10 (41.7%)
Line related complications	3 (12.5%)
Platelet transfusion needed for line insertion	11 (45.8%)
Infection	4 (16.7%)
*Isolated organisms:*	
• Acetinobactor	1 (4.2%)
• Klebsiella	1 (4.2%)
• Pseudomonas	1 (4.2%)
Remission Achieved	21 (87.5%)
Alive	20 (83.3%)
Dead	4 (16.7%)
	One in CR

**Fig.1:**
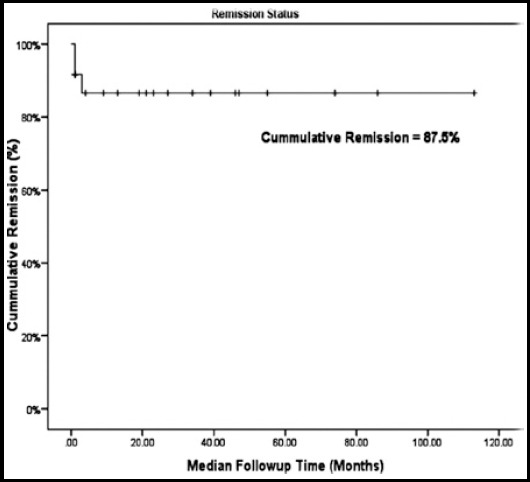
Remission status of TTP patients over period of follow-up.

Twenty-one of 24 (87.5%) patients achieved complete remission (CR) without any subsequent relapse. On long-term median follow up of 22 months (Range 1-113), overall survival was 83.3%. Three patients died during acute episode because of very sever disease or delayed arrival to our center. One patient died later on because of other comorbidities while in CR (Fig.1).

## DISCUSSION

Although some publications include large number of TTP patient^12^,^13^ but only few case reports have evaluated the clinical features, laboratory parameters and therapeutic outcomes of TTP in Saudi patients.^9^-^11^ In this study, we analyzed records of 24 patients with TTP. Triad of thrombocytopenia, MAHA along with neurologic manifestations was present in (91.7%) of the patients. But, the classic pentad (thrombocytopenia, anemia, fever, neurologic and renal abnormalities) was present in only a minority of cases (8.3%). These results are similar to that reported by Radolfi et al.^14^ and suggest that this triad is an important clue for the diagnosis of TTP.

It has been well documented that a significant difference in incidence exists in different age groups and between the male and female sex.^15^,^16^ In our series, the incidence of TTP was evidently higher in women than in men with a female to male ratio of 3:1, and the median age was 33.5 years (range 17–63). Our observations suggest that sex and age distribution in Saudi TTP is similar to other populations of the world.^12^,^13^

TTP may be either congenital or acquired. Acquired factors observed in our series were mainly SLE-related (29.2%); pregnancy-related (16.66% including one with SLE and one with congenital TTP). Thirteen (54.16%) cases were classified as idiopathic; while one was congenital TTP in our series. The distribution of potential predisposing factors of TTP was thus slightly different from that reported by others.^17^,^18^

ADAMTS13, a plasma metalloproteinase, regulates platelet adhesion and aggregation through cleavage of VWF multimers. The importance of ADAMTS13 activity level in TTP is controversial.^18^,^19^ Coppo et al. showed that 71% of patients clinically diagnosed with TTP had severe ADAMTS13 deficiency.^20^ In Oklahoma registry data of 301 patients 13% of all patients, and 33% of patients with idiopathic TTP-HUS, had very severe ADAMTS-13 deficiency (<5% activity).^12^ The presenting features and clinical outcomes of patients with severe ADAMTS-13 deficiency were heterogeneous and not distinct.^12^

We determined the ADAMTS13 activity of 19 patients, and found that 8(42.1%) had very severe ADAMTS13 activity deficiency and four of them were idiopathic, 5(26.3%) had severe deficiency, 3(15.8%) had low activity, whereas 3(15.8%) had normal activity. In contrast Vesely et al.^5^ suggested that severe deficiency of ADAMTS13 activity may be a valuable indicator for idiopathic TTP. However, our result have demonstrated that even if ADAMTS13 activity was within the normal range, it was difficult to exclude the diagnosis of TTP. Slightly decreased ADAMTS13 activity is also reported in other clinical conditions, like DIC, SLE, liver cirrhosis, ITP and others.^15^,^21^-^23^ Overall, these and our observations provide a reasonable explanation why perplexing clinical scenarios occur in which decreased ADAMTS13 activity level does not parallel the clinical symptoms of TTP.^13^

Inhibitory antibodies against ADAMTS13 have been reported in 44-93% of TTP patients;^9^ and among our 9 tested patient inhibitor was detected in 6 (66.6%) patients with a titer range of 0.425 BU.

All our patients received TPE. On Day 1 and Day 7 of TPE, Median platelet count, hemoglobin and LDH had significant differences (P value< 0.001, < 0.007 and < 0.001 respectively) that reflects efficacious therapy given. Five patients (20.8%) were early refractory to TPE. Thirteen (54.2%) patients received rituximab either due to refractoriness to TPE on day 5-7, cardiac or neurological involvement (at treating physician’s discretion). Median number of TPE sessions 15.5(1–52) and median volume of plasma exchanged/day was 1.2(1-1.5). Number of mean TPE sessions in early rituximab group (who received rituximab within five days) was 13.5 while it was 17.4 in those who received later than day 5 (showing some trend). Our findings confirm the initial Phase II study done by Scully et al.^19^ In our patients, 87.5% of cases achieved complete remission and on long-term follow up of 22 months (1-113), 83.3% patient are alive. Three patients died during acute episode because of severe disease whereas one patient died later on due to comorbidities. There is not even a single relapsed observed so far. This is in contrast to many studies stating relapse rate up to 30-50%.^5^,^24^ No long-term relapse is extraordinary finding in our series and may be related to use of Rituximab in 13(54.2%) patients. There were no serious immediate complications of rituximab and on longer term follow up, we have not found any case of leukoencephlopathy.

TPE is frequently associated with adverse events.^25^ Among our 24 patients 3(12.5%) had TPE-associated complications: one each of hemorrhage, accidental removal and line blockage. Eleven (45.8%) required platelet transfusion prior to line insertion and 4(16.7%) developed infection with Acinetobacter, Klebsiella pneumoniae, or Pseudomonas.

On admission, 10(41.7%) of the patients required ICU support because of altered conscious level, or critical lab values justifying ICU admission. This finding is similar to another study on TTP by Scully et al.^19^

We conclude that in spite of the fact that TTP is a life threatening condition, immediate treatment with TPE along with steroids and or rituximab was very effective in preventing high risk of mortality and achieving durable CR in 87.5% of our patients. Combination of very severe CNS manifestations and delayed arrival contributed to mortality significantly. More awareness is needed for early diagnosis and early referral to higher centers.
